# Clinical outcomes of plastic versus self-expanding metal stents in malignant hilar biliary obstruction

**DOI:** 10.1007/s00464-026-13099-4

**Published:** 2026-07-08

**Authors:** Pastor Joaquín Ortiz Mendieta, Bruno da Costa Martins, Deborah Marques Centeno, Julia Mayumi Gregorio, Rafael Utimura Sueta, Adriana Vaz Safatle Ribeiro, Caterina Maria Pia Simoni Pennacchi, Carla Cristina Gusmon, Gustavo Andrade de Paulo, Luciano Henrique Lenz Tolentino, Marcelo Simas de Lima, Renata Nobre Moura, Sebastian Naschold Geiger, Fauze Maluf-Filho

**Affiliations:** Division of Endoscopy, Instituto do Câncer do Estado de São Paulo, Department of Gastroenterology of Universidade de São Paulo, Av. Dr. Arnaldo, 251, Cerqueira César, São Paulo, SP 01246-000 Brazil

**Keywords:** Endoscopy, Biliary tract neoplasms, Cholestasis, Stents

## Abstract

**Background:**

Malignant hilar biliary obstruction (MHBO) is a challenging condition with a poor prognosis, often requiring palliative biliary drainage. Endoscopic placement of plastic stents (PS) or self-expanding metal stents (SEMS) are the preferred approaches, but the optimal choice remains under debate. This study compares the clinical outcomes, patency, and survival associated with PS and SEMS in patients with unresectable MHBO.

**Methods:**

This retrospective study analyzed patients with MHBO who underwent endoscopic drainage between January 2015 and December 2022 at a tertiary cancer center. Clinical success, stent patency, survival, and reintervention rates were compared between patients receiving PS or SEMS. Cox proportional hazards models and Kaplan–Meier survival analyses were used to evaluate outcomes.

**Results:**

A total of 86 patients were included, with 39 (45.3%) receiving SEMS and 47 (54.7%) receiving PS. Clinical success was higher in the SEMS group (76.9% vs. 51.1%; p = 0.015), but this association was not confirmed in multivariate analysis. SEMS demonstrated significantly longer patency (median 213 vs. 78 days; p = 0.01) and a lower risk of recurrent biliary obstruction (HR 3.17, 95% CI 1.60–6.27; p = 0.001). However, overall survival did not differ significantly between groups (p = 0.059). Multivariate analysis identified better ECOG status (0–2) and bilirubin levels < 10 mg/dL as independent predictors of clinical success and longer survival.

**Conclusion:**

SEMS provide superior patency compared to PS but did not significantly impact overall survival. Clinical success was influenced by ECOG performance status and baseline bilirubin levels rather than stent type alone.

Malignant hilar biliary obstruction (MHBO) has a dismal prognosis, with an estimated 5-year survival rate of less than 10% [1, 2]. It arises from various malignancies, including hilar cholangiocarcinoma, hepatocellular carcinoma, intrahepatic cholangiocarcinoma, gallbladder cancer, and metastases [1, 3, 4]. Patients typically present with cholestatic jaundice [1, 5]. Due to early nonspecific symptoms, most individuals (around 73%) [1] are diagnosed at an advanced stage, limiting the potential for curative resection [1, 6–9].

For patients with advanced MHBO who are not candidates for surgical resection or neoadjuvant chemotherapy, palliative chemotherapy is often indicated to extend survival. However, recurrent biliary obstruction (RBO) after stenting for malignancy can compromise treatment, and lead to life-threatening complications such as acute cholangitis [7, 10]. Therefore, the effective management of jaundice and the prevention of cholangitis are essential for continued chemotherapy [6, 11, 12].

Endoscopic drainage using stent placement is the preferred palliative approach because it is less invasive than surgical or percutaneous alternatives [5, 13]. However, there is no consensus on the ideal drainage strategy or the best type of stent [1, 3, 14]. Among the available options, plastic stents (PS) and uncovered self-expanding metallic stents (SEMS) are commonly used [15, 16]. The ideal treatment should not only relieve jaundice effectively but also provide sustained patency [7, 10, 14].

While many studies have shown that SEMS result in lower dysfunction rates and longer patency compared to PS, their benefits and impact on survival remain controversial. Some studies report improved clinical success and survival rates with SEMS [3, 8], but these findings have not been consistently replicated [6, 9, 17]. Although there is general agreement that SEMS offer longer patency and reduced dysfunction, one study highlighted a higher incidence of percutaneous transhepatic biliary drainage (PTBD) in patients with SEMS [9].

Advances in systemic treatments, such as chemotherapy, immunotherapy, and targeted therapies, have contributed to longer survival in patients [18–20], potentially increasing the need for additional interventions due to recurrent bile duct obstructions. Therefore, in the context of reintervention, it is crucial to consider the risk of drainage failure, which may demand PTBD.

## Objective

The primary aim of this study was to assess the clinical success and stent patency rates of endoscopic drainage using SEMS versus PS in patients with unresectable malignant hilar biliary obstruction. Secondary objectives included overall survival, reintervention rates and the need for PTBD.

## Materials and methods

### Study design

This retrospective study was conducted at the Instituto do Câncer do Estado de São Paulo (ICESP). Data on endoscopic retrograde cholangiopancreatography (ERCP) procedures were retrieved from the ERCP database for malignant neoplasms. The study was approved by the Ethics Committee of our institution (Scientific Teaching and Research Committee – CCEP 4071/23) and was registered in the National Database for Human Research (Plataforma Brasil—CAAE 70246623.1.0000.0068).

### Patients

Patients with unresectable MHBO classified as Bismuth II to IV who underwent endoscopic drainage between January 2015 and December 2022 were included in the study. Informed consent was obtained from all patients prior to the procedure. They were followed from the date of the first procedure at our institution until the date of death or until December 2023. Patients with drainage failure were excluded; this was defined as technical failure of the procedure, including inability to access the bile duct, inability to cross biliary stenosis, or unsuccessful stent deployment. Patients who had simultaneous placement of plastic and metal stents were also excluded.

Whenever possible, neoplasm histology was obtained based on brush cytology or biopsies taken during ERCP, endoscopic ultrasound-guided fine-needle aspiration (EUS-FNA), percutaneous puncture or surgery. In patients without tissue evidence of malignancy, the diagnosis was based on imaging (computed tomography, magnetic resonance imaging) combined with clinical history and outcomes during follow-up.

### Procedure

ERCP was performed by advanced endoscopy fellows under the supervision of experienced endoscopists, with > 200 ERCP per year. All procedures were performed under general anesthesia.

After successful biliary cannulation and positioning of the guidewire across the stricture, bile was aspirated to decompress the obstructed duct, followed by contrast injection. Biliary sphincterotomy was performed at operator’s discretion. When possible, two or more guidewires were introduced to perform selective drainage of different segments.

The type, diameter and number of stents were defined according to the decision of the endoscopist, based on anatomical complexity, technical considerations, and overall patient condition, with preference for the most dilated segments and preserved liver parenchyma. Drainage of ≥ 50% of the liver was generally attempted, or at least 33%, in patients with preserved liver function. If drainage of more than one sector was not possible, the drainage of only one was attempted, prioritizing the largest portion of liver volume. The target lobes to be drained were decided by the operators according to the cross-sectional image evaluated before the procedure. Drainage of atrophic segments or segments diffusely occupied by the tumor or metastasis was avoided. If necessary, before stent insertion, the hilum stenosis was dilated with a dilation bougie (Cook Medical, Bloomington, IN, USA) and/or a balloon (QBD 4 mm or 6 mm, Cook Medical, Bloomington, IN, USA).

Reintervention was performed in symptomatic patients with RBO. In patients with good performance status and controlled disease on cross-sectional imaging, a scheduled stent replacement before RBO was also performed. During endoscopic reinterventions, plastic stents were removed and new plastic or metallic ones were placed. In the case of metal stent dysfunction, plastic stents or a new uSEMS were placed inside the previous SEMS. Patients who failed endoscopic reintervention were referred for PTBD. EUS-guided hepaticogastrostomy was not part of the management algorithm of these patients.

Straight plastic stents of 7, 8.5 or 10 Fr were used (Advanix biliary stents—Boston Scientific Corporation, MA, USA). The length was chosen based on the location of the stenoses. SEMS were uncovered, 8–10 mm diameter, 8–10 cm long, WallFlex Biliary RX Uncovered Stent (Boston Scientific Corporation, MA, USA), Biliary stent Non-Covered (Micro-Tech, Nanjing, China) and Hanarostent® Biliary (Olympus Corporation, Shinjuku, Tokyo, Japan).

### Definitions

Technical success was defined as the successful implantation of single or multiple stents, through the biliary stenosis, with drainage of at least one hepatic sector [19]. Clinical success was considered when the serum bilirubin level decreased to normal (≤ 1.2 mg/dL) or there was a ≥ 50% reduction in the value prior to stent placement, within 14 days of drainage, without further intervention [4, 9, 19]. Recurrent biliary obstruction was defined as stent dysfunction due to occlusion or migration, causing an increase in serum bilirubin and/or clinical symptoms as cholangitis [19]. Stent patency was measured from initial placement to RBO or patient death. Reintervention was defined as any type of endoscopic or percutaneous procedure necessary to improve biliary drainage after RBO. Overall survival was defined as the time from the first endoscopic stent placement to the moment of death.

### Statistical analysis

Quantitative variables were assessed using either unpaired Student’s t-tests (for parametric data) or Mann–Whitney U tests (for non-parametric data). Qualitative or categorical variables were described as simple frequencies and percentages, and group comparisons were made using the Chi-square test or Fisher’s Exact test when appropriate. Survival and stent patency were analyzed using Kaplan–Meier curves, with the log-rank test applied to independent variables such as sex, age, stenosis level, Eastern cooperative oncology group scale (ECOG), stent type, bilirubin level, cholangitis. Multivariate analysis for clinical success (dependent variable) was performed using binary logistic regression, considering independent variables such as gender, age, stenosis level, ECOG, stent type, bilirubin level, and cholangitis. Proportional hazards Cox regression was employed for survival and patency outcomes, with the same covariates. Statistical significance was defined as p ≤ 0.05. All analyses were conducted using SPSS version 22.0.

## Results

**Fig. 1:**
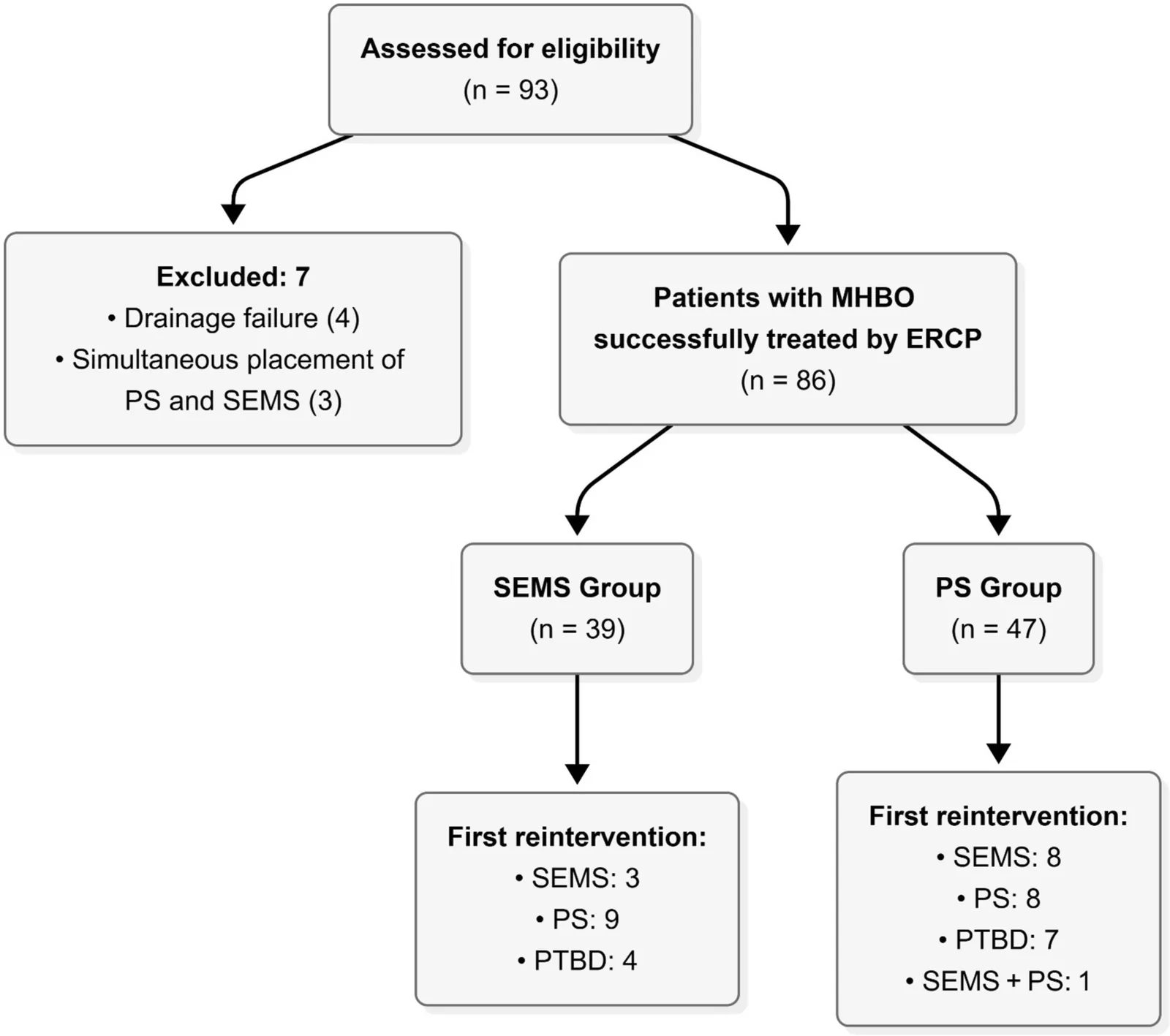
Patient flow diagram

We retrospectively evaluated 93 patients, of whom 7 were excluded (4 due to drainage failure and 3 who were treated simultaneously with plastic and metal stents). Finally, 86 patients successfully treated by ERCP were included for analysis (31 men and 55 women). Of these, 34 (39.5%) had a diagnosis of hilar cholangiocarcinoma, 44 (51.16%) had metastatic tumors, and 8 (9.31%) had gallbladder cancer. A total of 39 patients received SEMS, while 47 received PS. The groups were similar at baseline, except for a higher number of patients with ECOG 0–2 in the SEMS group compared to the PS group (p = 0.043). Figure 1 presents the study flowchart, and the baseline characteristics of the patients are summarized in Table 1.

**Table 1:** Baseline characteristics of the study population

Variable	Category	Stent		Total (n)	p-value
		SEMS (n = 39)	PS (n = 47)		
Age, years (n, %)	< 60	22 (52.4)	20 (47.6)	42	0.279
	≥ 60	17 (38.6)	27 (61.4)	44	
Sex (n, %)	F	23 (41.8)	32 (58.2)	55	0.499
	M	16 (51.6)	15 (48.4)	31	
Bismuth classification (n, %)	II	14 (60.9)	9 (39.1)	23	0.233
	III	10 (38.5)	16 (61.5)	26	
	IV	15 (40.5)	22 (59.5)	37	
Aetiology (n, %)	Cholangiocellular carcinoma	14 (41.2)	20 (58.8)	34	0.583
	Gallbladder cancer	5 (62.5)	3 (37.5)	8	
	Metastatic tumor	20 (45.5)	24 (54.5)	44	
ECOG (n, %)	0–2	34 (51.5)	32 (48.5)	66	0.043
	3–4	5 (25)	15 (75)	20	
Bilirubin level (n, %)	< 10	22 (53.7)	19 (46.3)	41	0.193
	≥ 10	17 (37.8)	28 (62.2)	45	
Cholangitis (n, %)	No	32 (48.5)	34 (51.5)	66	0.318
	Yes	7 (35)	13 (65)	20	

*ECOG* Eastern cooperative oncology group scale, *SEMS* self-expanding metal stents, *PS* plastic stents

### Comparison of outcomes between the type of stents (SEMS vs PS)

Clinical success was observed in 76.9% of patients in the SEMS group, compared with 51.1% in the PS group (p = 0.015). Reintervention to restore biliary drainage was required in 16 patients (41.02%) in the SEMS group and 24 patients (51.06%) in the PS group. The technique used for the first reintervention is summarized in Fig. 1.

Mean time to reintervention was significantly longer in the SEMS group than in the PS group (132.4 ± 129.4 days vs. 66.7 ± 85.7 days, respectively; p = 0.028). In addition, a significantly higher proportion of patients in the SEMS group received chemotherapy following biliary drainage (61.5% vs 29.8%; p = 0.003).

There was no statistically significant difference between the groups regarding median overall survival (179 days [95% CI 83.6–274.4] vs. 98 days [95% CI 46.5–149.5]; p = 0.059), the mean number of endoscopic reinterventions (0.92 vs. 0.79; p = 1.00), total reinterventions (1.15 vs. 1.00; p = 0.807), or the need for PTBD (23.1% vs. 40.4%; p = 0.087). These results are summarized in Table 2.

**Table 2:** Comparison of clinical outcomes between SEMS and plastic stents

	SEMS (n = 39)	PS (n = 47)	p-value
Clinical success (n, %)	30 (76.9%)	24 (51.1%)	0.015
Endoscopic reinterventions (mean ± SD)	0.92 (± 1.77)	0.79 (± 1.41)	1.000
Total reinterventions (mean ± SD)	1.15 (± 1.84)	1.00 (± 1.44)	0.807
Time to reintervention, days (mean ± SD)	132.4 (± 129.4)	66.7 (± 85.7)	0.028
Median survival, days (95% CI)	179.0 (83.6–274.4)	98.0 (46.5–149.5)	0.059
PTBD (n, %)	9 (23.1%)	19 (40.4%)	0.087
Chemotherapy post-drainage (n, %)	24 (61.5%)	14 (29.8%)	0.003

*PTBD* percutaneous transhepatic biliary drainage, *SEMS* self-expanding metal stents, *PS* plastic stents

**Table 3:** Association between baseline characteristics and clinical success

Variable	Category	Clinical success		Total (n = 86)	p-value
		No	Yes		
Age, years (n, %)	< 60	14 (33.3)	28 (66.7)	42	0.509
	≥ 60	18 (40.9)	26 (59.1)	44	
Gender (n, %)	F	21 (38.2)	34 (61.8)	55	0.821
	M	11 (35.5)	20 (64.5)	31	
Bismuth classification (n, %)	II	6 (26.1)	17 (73.9)	23	0.019
	III	6 (23.1)	20 (76.9)	26	
	IV	20 (54.1)	17 (45.9)	37	
ECOG (n, %)	0–2	19 (28.8)	47 (71.2)	66	0.004
	3–4	13 (65)	7 (35)	20	
Stent (n, %)	SEMS	9 (23.1)	30 (76.9)	39	0.015
	PS	23 (48.9)	24 (51.1)	47	
Bilirubin level, mg/dL (n, %)	< 10	9 (22)	32 (78)	41	0.007
	≥ 10	23 (51.1)	22 (48.9)	45	
Cholangitis (n, %)	No	26 (39.4)	40 (60.6)	66	0.599
	Yes	6 (30)	14 (70)	20	

*ECOG* Eastern cooperative oncology group scale, *SEMS* self-expanding metal stents, *PS* plastic stents

### Clinical success

Patients with Bismuth II and III stenoses demonstrated significantly higher clinical success rates compared to those with Bismuth IV (p = 0.019). Similarly, patients with better performance status (ECOG 0–2) showed greater clinical success than those with ECOG 3–4 (71% vs. 35%, p = 0.004). Clinical success was also more frequent among patients who received SEMS (p = 0.015) and those with baseline bilirubin levels below 10 mg/dL (p = 0.007). No significant associations were found between clinical success and age, gender, or the presence of cholangitis (Table 3).

Univariate analysis corroborated the findings of the Chi-square analysis, reinforcing the associations between clinical success and Bismuth classification, ECOG performance status, stent type, and baseline bilirubin levels. Additionally, for each one-point increase in pre-drainage bilirubin level, the likelihood of clinical success decreased by 8% (OR 0.92, 95% CI 0.86–0.99; p = 0.021).

In the multivariate analysis, however, SEMS was not associated with clinical success (OR 0.43, 95% CI 0.14–1.29; p = 0.131). Conversely, Bismuth II or III obstruction levels, ECOG 0–2, and lower pre-drainage bilirubin levels were independently associated with higher clinical success rates. Table 4 presents the results of binary logistic regressions for clinical success.

**Table 4:** Multivariate logistic regression analysis for clinical success

Variable	Category	Univariate		Multivariate	
		OR (95% CI)	p-value	OR (95% CI)	p-value
Age (years)	< 60	1.00			
	≥ 60	0.72 (0.30–1.74)	0.468		
Gender	F	0.89 (0.36–2.22)	0.804		
	M	1.00			
Bismuth classification	II	3.33 (1.07–10.35)	0.037	4.58 (1.28–16.36)	0.019
	III	3.92 (1.28–12.0)	0.017	5.36 (1.48–19.36)	0.010
	IV	1.00		1.00	
ECOG	0–2	1.00		1.00	
	3–4	0.22 (0.08–0.63)	0.005	0.18 (0.06–0.61)	0.006
Stent	SEMS	1.00		1.00	
	PS	0.31 (0.12–0.80)	0.015	0.43 (0.14–1.29)	0.131
Cholangitis	No	0.66 (0.22–1.93)	0.448		
	Yes	1.00			
Bilirubin level		0.92 (0.86–0.99)	0.021	0.92 (0.86–1.00)	0.038

*ECOG* Eastern cooperative oncology group scale, *SEMS* self-expanding metal stents, *PS* plastic stents, *OR* odds ratio, *CI* confidence interval

### Patency

**Fig. 2:**
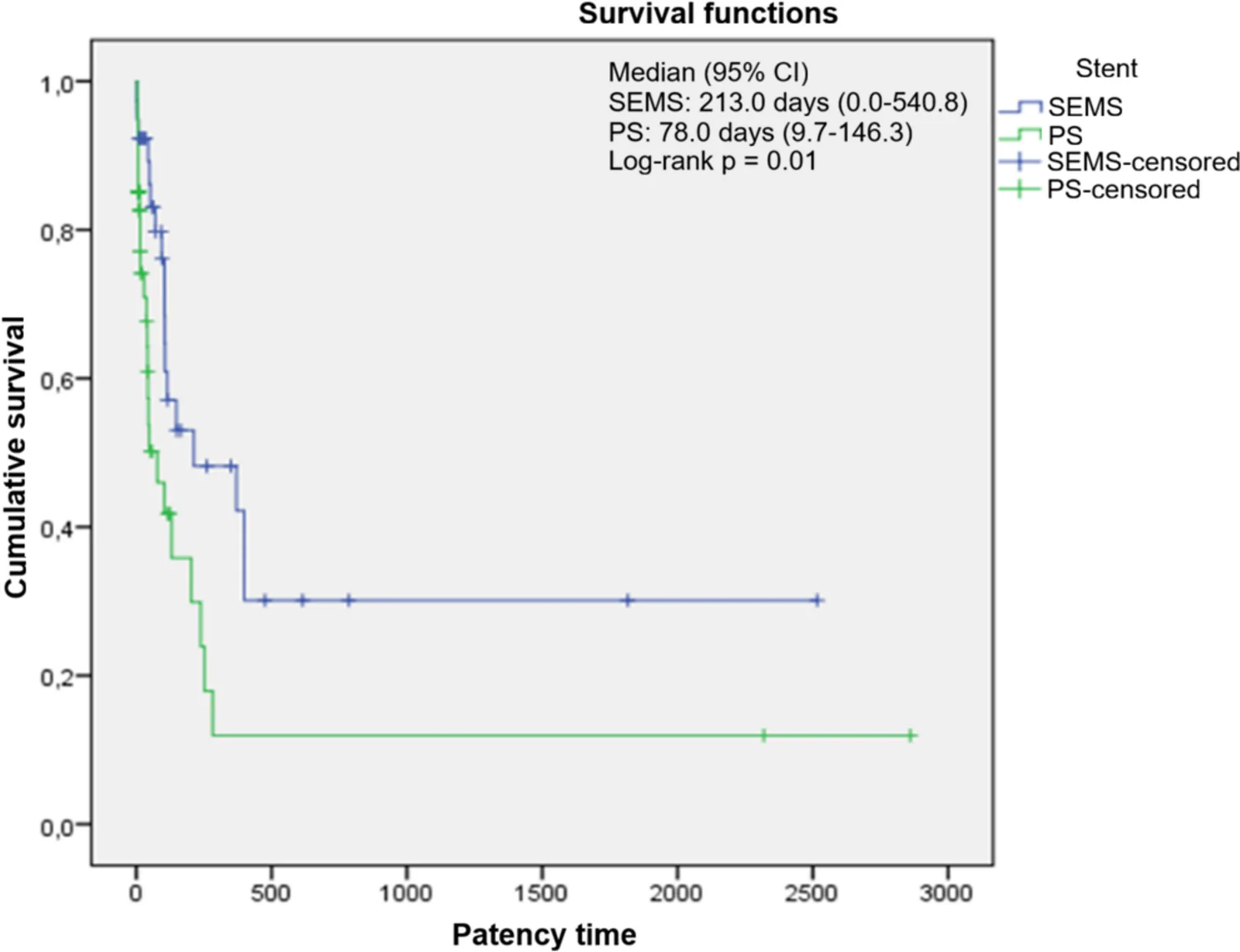
Kaplan–Meier analysis of stent patency

Kaplan–Meier curves and the log-rank test were applied to the categorical variables: age, gender, Bismuth level, ECOG score, and type of stent (plastic versus metallic). SEMS demonstrated significantly higher patency rates, as determined by the log-rank test (p = 0.01) (Fig. 2). No significant differences were found between the groups for the other tested variables.

The univariate Cox regression analysis showed that the PS group had a HR of 2.22 (95% CI 1.19–4.12; p = 0.012), indicating higher chances of recurrent biliary obstruction compared to the SEMS group. This finding was confirmed by multivariate analysis, with an HR of 3.17 (95% CI 1.60–6.27; p = 0.001) (Table 5).

**Table 5:** Cox proportional regression analysis for stent patency

Variable	Category	Univariate		Multivariate	
		HR (95% CI)	p-value	HR (95% CI)	p-value
Age (years)	< 60	1.00		1.00	
	≥ 60	0.76 (0.42–1.40)	0.384	0.55 (0.29–1.04)	0.065
Gender	F	1.21 (0.64–2.30)	0.555		
	M	1.00			
Bismuth classification	II	0.97 (0.46–2.05)	0.941		
	III	1.21 (0.60–2.46)	0.596		
	IV	1.00			
ECOG	0–2	1.00		1.00	
	3–4	0.62 (0.26–1.48)	0.284	0.43 (0.18–1.06)	0.067
Stent	SEMS	1.00		1.00	
	PS	2.22 (1.19–4.12)	0.012	3.17 (1.60–6.27)	0.001
Cholangitis	No	0.81 (0.42–1.58)	0.541		
	Yes	1.00			
Bilirubin level		1.03 (0.98–1.08)	0.234	1.04 (0.99–1.09)	0.116

*ECOG* Eastern cooperative oncology group scale, *SEMS* self-expanding metal stents, *PS* plastic stents, *HR* hazard ratio, *CI* confidence interval

### Survival

**Fig. 3:**
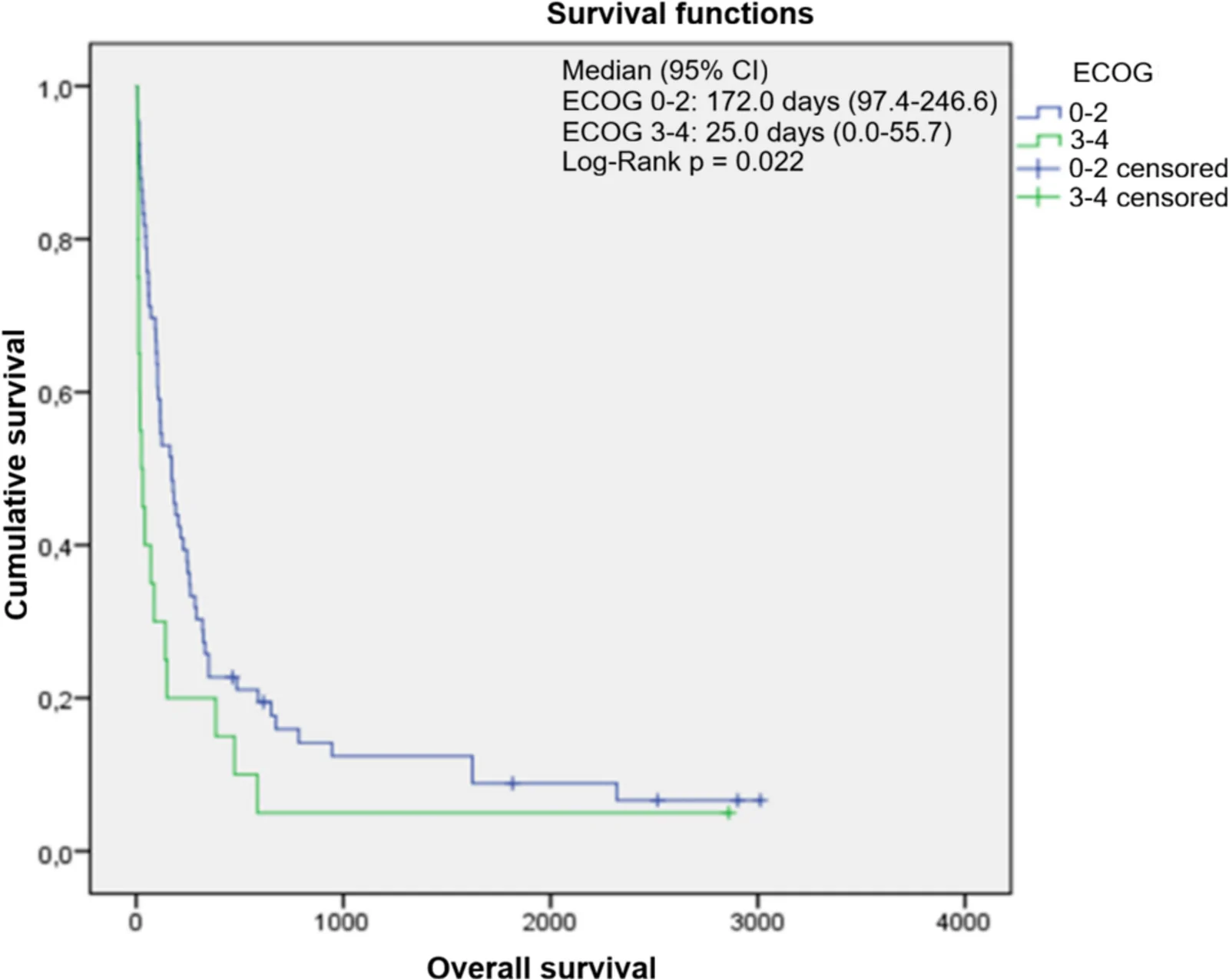
Kaplan–Meier analysis of overall survival according to ECOG performance status

**Fig. 4:**
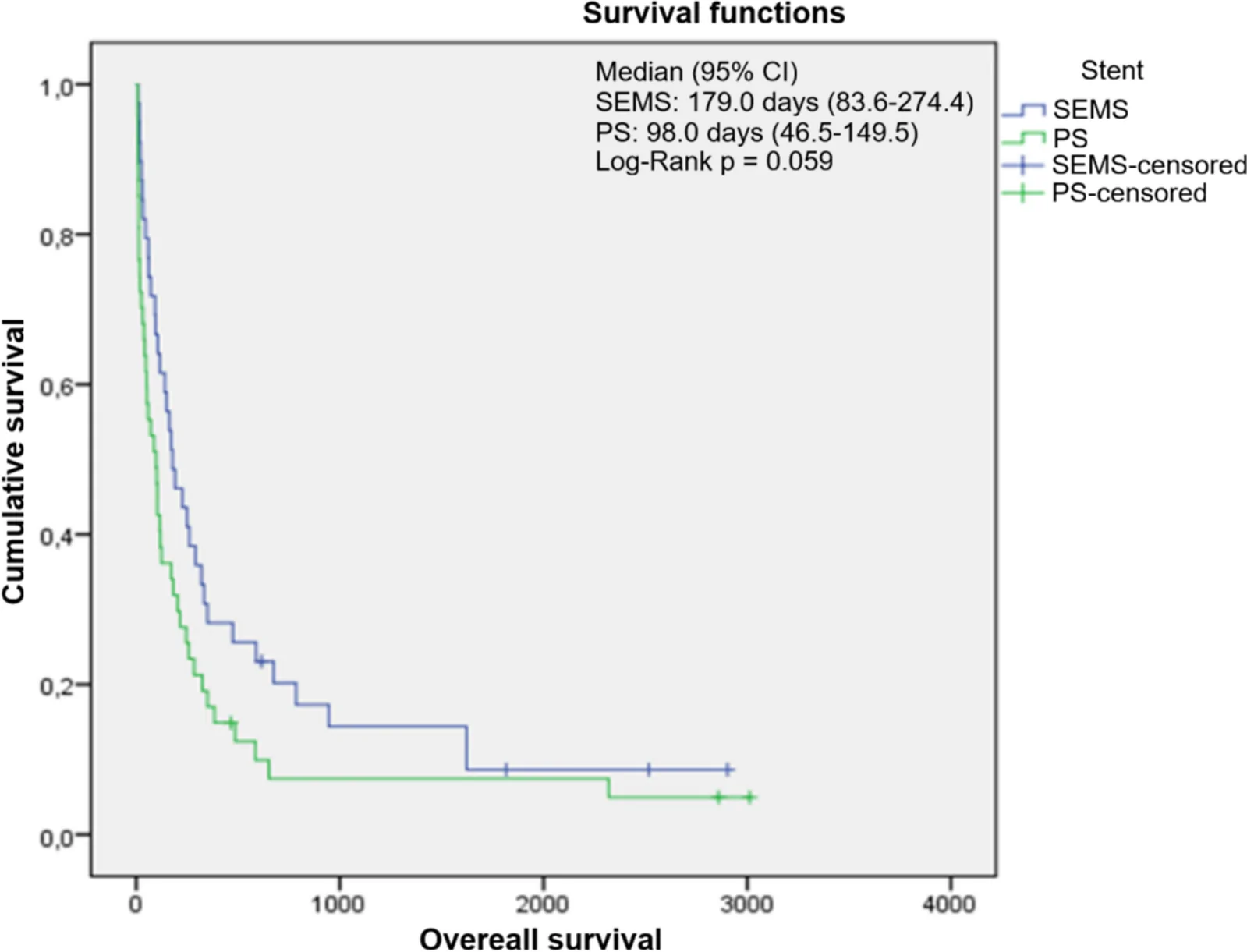
Kaplan–Meier analysis of overall survival according to stent type

**Fig. 5:**
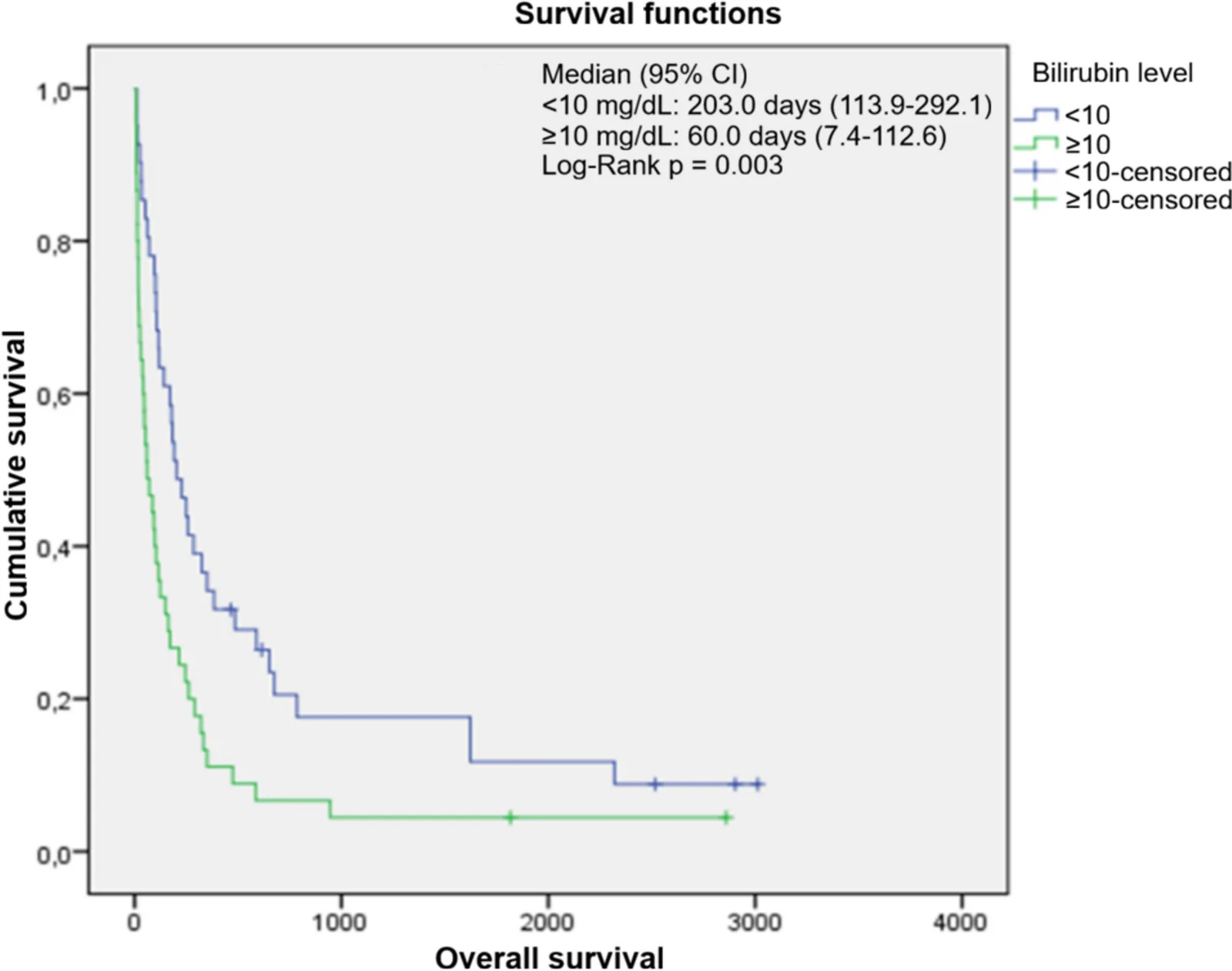
Kaplan–Meier analysis of overall survival according to baseline bilirubin level

The log-rank test showed higher survival rates (p = 0.022) for patients with better performance status (ECOG 0–2), and for those with bilirubin levels < 10 mg/dL (p = 0.003). Patients with SEMS showed a marginal, non-statistically significant difference in survival compared to those with plastic stents (p = 0.059) (Figs. 3, 4 and 5).

At univariate Cox regression analysis, patients with ECOG 3–4 had a 1.81 times higher risk of death compared to those with ECOG 0–2 (95% CI 1.08–3.05; p = 0.025). Additionally, for each unit increase in bilirubin, the risk of death increased by 6% (95% CI 1.02–1.09; p = 0.002). Patients with PS had a 1.53 times higher risk of death compared to those with SEMS, although this difference was marginal and not statistically significant (95% CI 0.98–2.40; p = 0.062). In multivariate analysis, ECOG and bilirubin levels were confirmed as significant risk factors (Table 6).

**Table 6:** Cox proportional regression analysis for overall survival

Variable	Category	Univariate		Multivariate	
		HR (95% CI)	p-value	HR (95% CI)	p-value
Age (years)	< 60	1.00			
	≥ 60	1.08 (0.69–1.68)	0.743		
Gender	F	1.04 (0.66–1.65)	0.865		
	M	1.00			
Bismuth classification	II	1.16 (0.66–2.02)	0.604		
	III	1.17 (0.69–1.99)	0.557		
	IV	1.00			
ECOG	0–2	1.00		1.00	
	3–4	1.81 (1.08–3.05)	0.025	1.77 (1.05–2.98)	0.033
Stent	SEMS	1.00			
	PS	1.53 (0.98–2.40)	0.062		
Cholangitis	No	1.23 (0.72–2.08)	0.453		
	Yes	1.00			
Bilirubin level		1.06 (1.02–1.09)	0.002	1.05 (1.02–1.09)	0.003

*ECOG* Eastern cooperative oncology group scale, *SEMS* self-expanding metal stents, *PS* plastic stents, *HR* hazard ratio, *CI* confidence interval

## Discussion

This study evaluated the clinical success, stent patency, and survival outcomes of self-expanding metal stents compared to plastic stents in the palliative treatment of malignant hilar biliary obstruction. We found that SEMS demonstrated superior patency, with a significantly lower risk of recurrent biliary obstruction compared to PS. However, clinical success and survival outcomes did not differ significantly between the groups. These results align with previous studies supporting the prolonged patency of SEMS while challenging the notion of a definitive survival advantage.

Two previous studies reported higher clinical success rates with SEMS (Xia: 99% vs. 71%; Sangchan: 70.4% vs. 46.3%) [3, 8], while two others found no significant difference [6, 9]. In our study, although SEMS was associated with higher clinical success in the univariate analysis (76.9% vs. 51.1%; p = 0.015), this association did not remain significant after multivariate adjustment. These findings suggest that the apparent benefit observed in the univariate model was likely influenced by baseline differences between groups, particularly the higher proportion of patients with favorable ECOG performance status in the SEMS group.

In contrast, Bismuth II and III classification, ECOG performance status, and baseline bilirubin levels below 10 mg/dL remained independently associated with clinical success, reinforcing the importance of disease extent and patient condition over stent type alone. Notably, for each one-unit increase in bilirubin level, the likelihood of clinical success decreased by 8%.

A similar pattern was observed in the survival analysis, where SEMS showed only a non-significant trend toward improved outcomes. These findings may reflect residual confounding as well as limited statistical power, given the relatively small sample size, and suggest that the impact of stent type on patient-centered outcomes is less pronounced than its effect on stent patency.

Notably, a higher proportion of patients in the SEMS group received post-drainage chemotherapy. This finding may be partially explained by more effective biliary drainage, potentially facilitating the initiation of systemic therapy. Nonetheless, this association should be interpreted with caution, as the greater prevalence of patients with better ECOG performance status in the SEMS group likely also contributed to the increased use of chemotherapy.

SEMS demonstrated superior patency compared to PS in Kaplan–Meier analysis (median 213 days vs. 78 days), a result confirmed in both univariate and multivariate analyses. This aligns with previous reports [3, 6, 9, 17], reinforcing that SEMS reduce the likelihood of early recurrent biliary obstruction. Given the higher dysfunction rates of PS, it was expected that these patients would require more reinterventions to maintain biliary drainage. Indeed, five prior studies have reported a higher number of reinterventions in patients receiving PS [3, 6, 9, 17, 21], while one found no significant difference [8]. However, no difference was observed in the total number of reinterventions or endoscopic reinterventions between groups. This apparent paradox may be explained by the longer survival observed in the SEMS group, which increases the cumulative time at risk for stent dysfunction and the need for subsequent interventions. Therefore, time to reintervention may be a more meaningful parameter than the absolute number of reinterventions when comparing stent performance.

A prior study [9] reported a higher need for PTBD in patients with SEMS (50% vs. 22.2% for PS); however, this was not replicated in our cohort, where no significant difference was observed. This suggests that the need for additional drainage procedures may be more dependent on patient-specific factors and disease progression rather than stent selection.

Regarding overall survival, a literature review identified two studies reporting longer survival with SEMS [3, 8], whereas three others found no significant difference between SEMS and PS [6, 9, 17]. SEMS was associated with a non-significant trend toward improved survival (p = 0.059). This apparent advantage may have been influenced by the more favorable baseline ECOG performance status observed in the SEMS group. Complementary analyses showed that survival was significantly associated with ECOG 0–2 and bilirubin levels below 10 mg/dL, with each one-point increase in bilirubin correlating with a 6% higher risk of death.

This study has several limitations. It was conducted at a single referral center, potentially limiting the generalizability of the findings to other institutions. Its retrospective design introduces potential selection bias, as stent choice was made at the discretion of the endoscopist, leading to possible confounding by indication. Patients in the SEMS group had better baseline ECOG performance status, a known predictor of clinical success and survival, which may have influenced the outcomes. Although multivariate analysis was performed, residual confounding cannot be excluded. In addition, the relatively small sample size, particularly in subgroup analyses, may have limited the statistical power to detect independent associations.

Effective biliary drainage in MHBO requires careful planning, often involving a multidisciplinary evaluation including interventional radiology, surgery, clinical oncology, and endoscopy. This procedure should ideally be performed in specialized centers with experienced personnel. Several factors should be considered when selecting the optimal stent, including tumor extent, anatomical involvement, expected survival, and quality of life.

## Conclusion

In patients with MHBO, SEMS demonstrated superior patency compared to PS although it did not significantly impact overall survival. Additionally, time to reintervention was significantly longer with SEMS, suggesting a clinically meaningful delay in recurrent biliary obstruction. Although univariate analysis suggested higher clinical success with SEMS, multivariate analysis did not confirm this association. Clinical success was more strongly associated with better ECOG performance status and pre-drainage bilirubin levels below 10 mg/dL, while Bismuth type IV classification predicted lower success rates.

Overall survival, the mean number of reinterventions, and the need for PTBD were comparable between the two groups. These findings highlight the importance of patient selection criteria beyond stent type when determining optimal biliary drainage strategies in MHBO.
